# Effect of Paula exercise method on functional outcomes of women with post fistula repair incontinence: a protocol for randomized controlled trial

**DOI:** 10.1186/s12905-021-01249-w

**Published:** 2021-03-09

**Authors:** Saratu Umar Aliyu, Shmaila M. Hanif, Isa Usman Lawal

**Affiliations:** 1 Department of Physiotherapy, Rasheed Shekoni Teaching Hospital, Dutse, Jigawa State, Nigeria; 2 Department of Physiotherapy, Faculty of Allied Health Sciences, College of Health Sciences, Bayero University, Kano, Nigeria; 3 Department of Health Professions, Faculty of Health, Psychology and Social Care, Manchester Metropolitan University, Manchester, England

**Keywords:** Vesicovaginal fistula, Post repair incontinence, Urinary incontinence, Paula exercise method, Pelvic floor muscle training

## Abstract

**Background:**

Post-fistula-repair incontinence (PFRI) is a common complication of vesicovaginal fistula (VVF) surgeries. It entails continuous leakage of urine after successful VVF closure. Pelvic Floor Muscle Training (PFMT) plays a vital role in the management of PFRI, however, an evolving exercise approach is the Paula Exercise Method (PEM) which has shown a promising effect in stopping urinary incontinence, but there is no data on its effect on PFRI. This study therefore, proposes to primarily investigate the effect of PEM on urine leakage and secondarily, pelvic floor strength (PFS), quality of life (QoL), sexual function (SF), and mental health (MH) in women with PFRI.

**Methods:**

This is a study protocol for a randomized controlled trial. A total of 182 participants are expected to participate in the study after satisfying the inclusion criteria. The participants will be randomized into either PEM or PFMT study groups. The demographic data of all the participants will be recorded. Each participant will be assessed for urine leakage, PFS, QoL, SF, and MH at baseline and subsequently, at four, eight and 12 weeks of intervention.

Demographic parameters will be summarized using descriptive statistics. Continuous data will be computed for differences using inferential statistic of Analysis of variance, t-test and Man Whitney U as appropriate. All analyses will be performed using SPSS version 22.0 with probability set at 0.05 alpha level.

**Discussion:**

It is hoped that the outcome of this study will determine the effect of the Paula exercise method on urine leakage, pelvic floor strength, quality of life, sexual function, and mental health among women with post-fistula-repair incontinence and also provide evidence for the use of the Paula method in urinary incontinence.

*Trial registration*: Pan African Clinical Trials Registry (www.pactr.org), identifier PACTR201906515532827.

## Background

Vesicovaginal fistula (VVF) is an abnormal opening between the vagina and the bladder that occurs often during childbirth as a result of prolonged obstructed labour [1, 2]. This occurs following compression on the pelvis by the foetal head resulting in tissue necrosis. This creates an opening between the vagina and bladder which leads to constant urine leakage from the vagina [1–3]. It is the major type of obstetric fistula, accounting for about 90% of all obstetric fistula cases [1, 4]. It is also regarded as one of the major causes of morbidity among females in developing countries and some parts of developed countries [2, 5]. The consequences of VVF can be devastating, as it affects women physically, socially, economically and psychologically [3, 4].

A number of studies have noted the risks for developing VVF as including: illiteracy, poverty or low socio-economic status, childbearing at an early age, lack of access to antenatal care, lack of skilled or trained traditional birth attendants, and a malformed pelvis [3–8]. Obstetric VVF affects 2 million women worldwide, with a prevalence of 3 per 1000 women in sub-Saharan Africa [2, 5, 8]. In Nigeria, according to the Federal Ministry of Health [9], there are over 20,000 new cases of VVF yearly, in addition to the approximately 400,000–800,000 existing cases awaiting surgical repair. Over 85% of all cases of VVF are found in the north-western part of Nigeria [7, 10]; high incidences are reported in Kano, Katsina and Bauchi states in this region of the country [7].

Surgical interventions have been proven to be the effective management for VVF, especially in complex fistula, and in small fistula if conservative treatment fails [1, 11]. Unfortunately, some women pass through the devastating period of having the VVF and undergoing the surgical procedure, but do not regain continence [1, 12, 13]. The VVF repair may be satisfactory anatomically, but inadequate functionally, as some women present with urinary leakage (called post-fistula repair incontinence) even after a successful repair [12–16].

Post-fistula repair incontinence (PFRI) as reported in the literature, is one of the most common complications of VVF surgeries, where urine continues to leak (especially with exertion) after successful VVF closure [12, 13, 17]. This incontinent gap is poorly understood and is a source of frustration to fistula surgeons (and particularly the patients) [1, 16, 18–20].

Risk for developing PFRI depends on the site and type of fistula, as well as pelvic floor strength (PFS). Women with severe pelvic floor muscle (PFM) weakness are at risk of developing PFRI [21, 22]. According to Kayondo et al., type IIb fistulae are six times more likely to cause incontinence after successful repair than type I fistulae [22]. Also, women with circumferential fistula or larg fistulas are less likely to be continent after surgery [23]. PFRI also arises due to small bladder size/capacity following loss of the surface area of the bladder wall due to extensive fistula, neurologic damage, or due to the involvement of the urethral sphincter, especially if there is marked tissue loss and scarring [1, 8, 12]. Furthermore, PFRI occurs if there is loss of the sphincteric mechanism of the vesicourethral junction, and reduced bladder capacity or injury to the urethra [14, 20]. It has been reported that the risk of PFRI is 50% in women with minimal vaginal scarring and good bladder volume, while is 100% in women with reduced bladder volume and/or severe vaginal scarring [22–24]. Conservative interventions are the mainstay in the treatment of PFRI [11, 14, 20]; but unfortunately, supplementary surgery is prioritized over non-surgical options; the consequence of this is further deterioration of the condition [24]. It has however, been suggested that surgery should only be considered in persistent cases of PFRI or if the conservative treatment failed [12, 13].

Physiotherapy plays a vital role in the management of urinary incontinence (UI) and its associated symptoms through rehabilitation of the pelvic floor muscles (PFM), either directly or indirectly. Directly, the PFM can be rehabilitated through: pelvic floor muscle training (PFMT), biofeedback, cone therapy, and neuromuscular electrical stimulation [25, 26]; and indirectly through exercising other muscles of the body like the transverse abdominal muscles and circular or ring muscles (known as Paula Exercise Method), and also by patient education [27–29]. However, there is limited data on the role of physiotherapy on PFRI specifically. The available data mainly used pelvic floor muscles training (PFMT) [8, 13, 14, 17, 30]. And, to achieve positive effects of the PFMT, the exercise needs to be individualized and performed under professional supervision [21]. In addition, women find it difficult to voluntarily contract their PFM if it is weak even with proper instructions, and instead they end up contracting their abdominal, gluteal, and hip adductor muscles, and try to exaggerate inspiration which will often worsen their condition. Moreover, most of the VVF centres do not have physiotherapists or trained continent nurses to guide the women in performing the correct PFMT [23]. Even if there are professionals, teaching and monitoring the PFMT of patients individually may not be possible due to the high number of victims. Therefore, there is the need to find a conservative approach that can be performed with more ease and possibly in a group to accommodate the large number of the affected women and to encourage peer support, to help the affected women to get back to their normal life and minimize the need for another surgery.

The Paula exercise method also called circular/ring muscle exercise is a growing conservative method used in the treatment of urinary incontinence (UI) in general. It is an easy-to-perform approach, and has been tested to be effective in the management of UI and its associated complications in women without previous fistula repair [27, 29–31]. Practically, the Paula exercise method focuses on contracting and relaxing specific circular (or ring) muscles of the body in an effort to rehabilitate other damaged muscles of the body, because the body sphincters works together and their activity can affect one another due to oscillations in the spinal cord [27, 28, 31, 32].

The Paula method is considered an alternative to PFMT and has been found to exhibit comparable efficacy for urinary incontinence [31, 33], including demonstrable long-term effects [34]. A study demonstrated comparable effectiveness of PFMT and PEM on sexual function and quality of life in women suffering from stress urinary incontinence [31]. However, a systematic review reported limited evidence for alternative exercise regimens for the reduction of urinary leakage in women with stress urinary incontinence [35]. The review found methodological limitations in PEM as an alternative to PFMT. The limitations included the fact that one of the studies was a pilot study [35] with small sample size while the other subjected the control, i.e. the PFMT group, to below-optimal training, and the high loss to follow-up (up to 28%). Additionally, both studies were conducted by the same group of researchers. These factors obviously suggest the need for more studies with robust methodological approaches in diverse groups of researchers from distinct settings to investigate the efficacy of the Paula exercise method. This study aims to contribute to filling this gap and is therefore, targeted at investigating the effectiveness of the Paula exercise method in women with PFRI.

## Study objectives

The main objective of this study is to investigate the effect of the Paula exercise method on urine leakage among women who suffered post-fistula repair incontinence (PFRI). In addition, the study will investigate the following:

1. Effect of the Paula exercise method on pelvic floor strength in women who suffered PFRI.
2. Effect of the Paula exercise method on quality of life of women who suffered PFRI.
3. Effect of the Paula exercise method on sexual function in women who suffered PFRI.
4. Effect of the Paula exercise method on mental health in women who suffered PFRI.

## Methods

### Study design and sample

The research design for this study is a randomized controlled trial (RCT). It is a quantitative research in the form of pretest, posttest and follow-up design. The study will be a two-arm, outcome assessor-blinded RCT. The two-arms will comprise an intervention and a control. Following participants’ consent and baseline assessments, eligible participants shall be allocated randomly into either the intervention group (Paula exercise method) or the control group (pelvic floor muscle training). The proposed timeline for this study is 12 calendar months from participants’ enrolment to follow-up assessments; Table 1 below:

**Table 1:** Study timeline from enrolment to completion

	Enrollment	Allocation	Post-allocation				Follow-up
Time point	− t	0	t0 (0 week)	t1 (4 weeks)	t2 (8 weeks)	t3 (12 weeks)	t4 (6 months)
Enrollment: eligibility screen informed consent allocation	X						
	X						
		X					
Intervention: Paula exercise (experimental group) Pelvic floor muscle training (control group)			X	X	X	X	
			X	X	X	X	
Assessments: baseline variables—age, weight, height, blood pressure, marital status, educational status, post fistula repair time	X	X					
Assessments: outcome variables [Primary outcome- urine leakage] [Secondary Outcome- pelvic floor strength, quality of life, sexual function, depression and anxiety]			X	X	X	X	X

### Setting

All participants will be recruited from Murtala Muhammad Specialist Hospital (MMSH) and Jahun General Hospital (JGH) in Kano and Jigawa states of Nigeria respectively. The training will be conducted at the physiotherapy departments of the two institutions. The MMSH is a major health institution in Kano State; it is a specialist and referral centre to all hospitals in Kano State and its neighbouring states. It has a bed capacity of eight hundred and twenty six (826), thirty (30) wards and units, nine (9) operating theatres, fourteen (14) clinics, and a staff strength of 1656. Similarly, JGH is a state hospital located in Jahun local government area of Jigawa State. It provides quality health services to people in Jigawa State and the neighbouring states. It is a 300-bed capacity hospital with diverse clientele who receive treatment from the different clinical departments of the facility.

### Participants

Participants in this study will comprise all women with PFRI attending the obstetrics and gynaecology departments of MMSH and JGH. However, only women who meet the study inclusion criteria will be screened and randomized into the experimental (Paula Exercise Method) and control (Pelvic Floor Muscles Training) groups. Participants will be screened for eligibility by the researcher and the research assistants. Participants will be considered eligible if: they have had the PFRI for more than 3 months, to ensure that healing has taken place [1, 14]; they present with a 1 g gain in weight following a pad test; they consent to participate through a written informed consent; they are not above the age of 50 because as women age changes occur in the bladder and pelvic structures that can increase the risk of developing incontinence [36]; they have no faecal leakage; they have not had previous rectal surgery; they are not receiving any other alternative PFM rehabilitation at the point of recruitment; and urinary infection is ruled out because it affects the integrity of the pelvic organs [37].

After screening for eligibility, a cough test will be used to differentiate the type of incontinence. In the cough test, if urine leakage coincides with the cough, it is classified as stress incontinence (SI), however, if the leakage is delayed till after the cough, it is urge incontinence. No leakage after the cough signifies that it is overflow and functional incontinence [38].

### Sample size

The sample size of this study was estimated based on the findings of a pilot study. We conducted a pilot study to test the adequacy and feasibility of this protocol as well as to determine the sample size required for this study. The pilot study participants were recruited after being certified as eligible to participate using the specified study inclusion criteria. Recruitment was conducted by reviewing records of the outpatient urology clinic on a weekly basis. A total of 30 women with post fistula repair incontinence participated in this pilot study. The 30 subjects were randomly assigned into the two arms of the study resulting into 15 participants in each arm of the pilot study (Paula Exercise Method and Pelvic Floor Muscle Training groups). All participants were assessed at baseline before randomization and prior to the commencement of intervention, post intervention (outcome). The primary outcome of interest is Urine leakage. The programme was structured for 4-weeks of daily routine activities in both groups. The detail of urine leakage assessment is described later in the manuscript as the primary outcome measure.

In this pilot study the mean age of participants was 29.9 ± 9.4, the median Urine leakage score for PEM and PFMT was 4.53 g and 21.61 g respectively. We computed Mann Whitney U for the data because the data was not normally distributed. Result for an independent two groups non-parametric test indicated significance (*U* = 38.00,* p* = 0.020) with an effect size of *Ƞ*^2^ = 0.32.

Using the outcome of the pilot study and according to a power calculation to detect a between-group difference, of 0.3 effect size for two independent means Mann Whitney U in urine leakage (UL) with 90% power at α = 0.05, a total sample size of 166 was generated. This suggests a total of 83 participants in each group. A 10% attrition rate will be considered, therefore, 182 participants will be recruited giving 91 participants in each group. The recruitment of participants from the two hospitals would be accomplished by: involving the surgeons from the departments of obstetrics and gynaecology of both hospitals who are involved in performing the fistula repairs (in the recruitment); and reviewing hospital registry and subsequently contacting patients whose records are available through individual phone contacts.

### Data collection procedure

This study has been approved by the Human Research Ethics Committees of Kano State and Jigawa State Ministries of Health.

Assessment of all participants will be carried out in four stages (at baseline, at 4 weeks, at 8 weeks, and at the end of the study). A comprehensive assessment of the participants will be conducted, where personal demographic information (age, height, weight, marital status, educational status) and PFRI-specific information (time since VVF repair, type/size of the VVF, amount of urine leakage) will be assessed at baseline; and functional status information (QoL, MH, SF, and PFS) will be assessed at baseline and after every four weeks up to 12 weeks. All the parameters will be assessed during follow-up after 6 months.

### Randomization

**Fig. 1:**
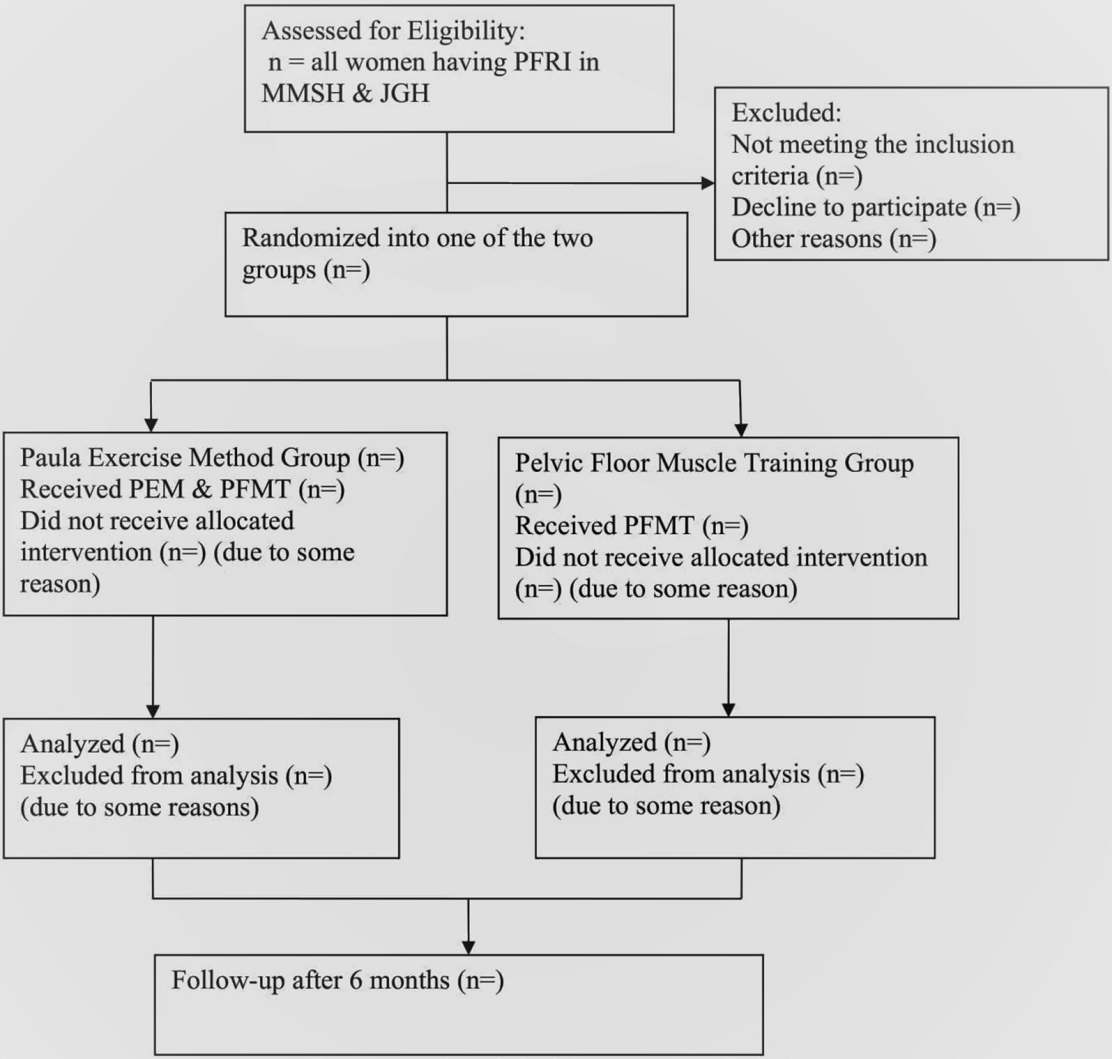
Study flow diagram

After screening the participants, and following baseline assessments, a computer-generated random allocation sequence will be used to assign the participants to either the intervention group (PEM group where they will receive PEM plus PFMT), or the control group (where they will receive PFMT only). To eliminate bias, the randomization will be carried out by a third party who is not aware of the study group. Assessment of outcome will be carried out by an experienced/trained researcher, who will be blinded to the type of intervention received by participants in each group. The study participants’ flow diagram is presented in Fig. 1

### Interventions

The interventions for the study will be PEM and PFMT. These training programmes will be administered by the researcher and four trained physiotherapists with postgraduate qualification in women’s health. Two research assistants will be attached to each of the two training groups (i.e. two for PEM and two for PFMT). All trainings in both groups will be carried out in the morning. Both training programmes are structured to conform to the Consensus on Exercise Reporting Template (CERT) checklist [39, 40].

## The Paula exercise method

The Paula exercise method will be adapted from previous studies where the method has been described in detail [27, 32]. No equipment will be needed for the exercise. The exercise will involve contracting and relaxing the eye muscles daily. The participants will first assume a comfortable sitting position on a chair (with back rest), and relax for two (2) minutes. The researcher or research assistance will teach the participant (until mastered) how to effectively contract (squeeze) the eye muscles and hold it for five (5) seconds, and then relax the muscles for three (3) seconds (this makes one exercise cycle). The exercise cycle will be repeated forty (40) times in a daily session. The same exercise procedure will be repeated daily by all the participants for twelve (12) weeks. The exercise procedure will be performed under the supervision of the researcher or any of the two research assistants during Week One of the study. Subsequently, participants will be instructed to continue with the exercise procedure at home for the remaining 11 weeks. Participants will each receive an activity log book in which to document their daily training activities (such as time training was performed and the duration of the activity) and any adverse event experienced during the training. The researcher and the research assistants will continually monitor the participants’ daily exercise performances through phone calls to ensure adherence. At four, eight and twelve weeks, each participant will be asked to visit the study centres (physiotherapy departments of either MMSH or JGH according to where the participant was recruited) to perform the exercise in group (according to the protocol specific for this group) in the presence of the researcher and/or research assistant and to re-assess the functional outcomes.

## Pelvic floor muscle training (PFMT)

The PFMT will be performed according to the clinical practice standard for pelvic floor exercise [27]. No equipment will be needed for the exercise procedure. The exercise will involve contracting (squeezing) and relaxing the PFM as if trying to stop urine from leaking or trying to raise the vagina up from the chair but, without contracting the abdominal, thigh, and buttock muscles. Each participant will be asked to comfortably lie supine on a bed (with knees in semi-flexed position) and relax for two (2) minutes before commencing the exercise. Each participant will receive detailed explanation on how to perform the exercise. Participants will then be asked to expose their vagina (to allow for visual assessment) and squeeze their PFM (as if trying to stop urination) and keep this position for a count of seven (7) and, then relax for a count of three (3). The researcher and/or the research assistant will observe the contraction of the PFM to ensure that the exercise is done correctly. Participants will be asked to use a mirror to accomplish biofeedback for better understanding and accuracy of the procedure [41]. The exercise will be repeated until mastered by the participant. The exercise will then be performed in a sitting position where each participant will assume a comfortable sitting position on a chair (with back rest) for two (2) minutes, and then perform the exercise procedure as explained above. The exercise will be performed forty (40) times during a session once daily. Participants will perform the procedure individually in the study centre (of either at MMSH or JGH) for the first week and under the supervision of the researcher and/or two (2) research assistants. Subsequently, participants will be asked to perform the exercise at home; the researcher or research assistants will track the participants’ individual daily exercise performance through phone calls to ensure adherence. Participants’ home-based activities (time and duration of training performed and any adverse event experienced) will be recorded daily in the activity log book which will be given to each participant. At four, eight and twelve weeks, each participant will visit the study centre (of either MMSH or JGH) to perform the exercise in group (according to the protocol specific for this group) in the presence of the researcher and/or research assistant and to re-assess the functional outcomes.

In both study groups, a participant’s treatment will be terminated/discontinued if the patient: (1) decides to opt out of the study, (2) develops persistent vaginal bleeding, (3) presents with other medical emergencies or adverse events including life-threatening episodes (uncontrollable bleeding); incapacitation; readmission into hospital or incidence of a sudden collapse resulting in a medical emergency within 48-h would be rated as severe adverse episodes. However, minor adverse events would include bruise due to a scratch by exercise equipment, fall that does not result in loss of consciousness, fracture, admission into hospital, dyspnoea, cut, muscle soreness or pain persisting beyond 48 h, and sudden hypertension or hypotension interfering with the study flow for a day. Participants’ relations would be asked to document any such events within the period of the study and/or report such situation to the research team. Regular medical/surgical care being received by the patients, not involving exercise procedure of similar characteristics to this study exercise interventions would be allowed to continue for participants in both groups.

### Outcome

A proforma will be developed by the researcher which will be used to document the demographic features (such as age, weight, height, blood pressure, address, type of incontinence, education status, and marital status) of all the participants as well as the pre, post and follow-up intervention findings of the study.

### Primary outcome measure

The primary outcome measure is urine leakage, and it will be assessed using the 1-h clinic pad test. The 1-h clinic pad test is an effective, simple, noninvasive, valid, and reliable method recommended by the International Continence Society for quantifying the amount of urine loss that will aid in the diagnosis/quantification of urinary incontinence, as well as assessing the efficacy of certain procedures that are used in the management of incontinence [38, 39]. The procedure for the pad test will be in line with the recommendation of the International Continence Society. Each participant will be asked to empty their bladder before the test, and drink 500 ml of water. A pre-weighed Always-Ultra pad will be given to the participant to place inside her underwear. The participant is then asked to sit for 15 min. After this, the researcher will instruct the participant to cough 10 times, squat 10 times, jump up and down on the spot 10 times, and walk briskly for 10 min. After 1-h, the pad will be removed and re-weighted using the Grams weighing scale. To quantify urine leakage, the difference in the pad weight will be calculated by subtracting the original pad weight from the pad weight after 1 h. A 1 g gain in pad weight will be considered as proof of UI, and a reduction in urine leakage to < 1 g after the intervention will be considered as a cure.

### Secondary outcome measures

The secondary outcome measures are Pelvic Floor Strength (PFS) to be measured using a perineometer; QoL to be measured using the Incontinence Quality of Life questionnaire; SF to be measured using the Female Sexual Function Index (FSFI); and Mental Health (MH) to be measured using the Hospital Anxiety and Depression rating scale (HADs). The perineometer is a highly reliable instrument used in assessing PFS with high intraclass correlation coefficient values of 0.95 and 0.88 for strength, and 0.94 and 0.83 for endurance, which indicate high within-day and between-days reliability for PFM strength and endurance respectively [42]. The PFS will be assessed according to standard protocol [42]. Each participant will be asked to lie supine with knees semi-flexed; the probe of the perineometer will be inserted into the vagina until the full extent of the compressible portion of the device is at the level of the hymen. Each participant would then be instructed to maximally contract her PFM by trying to pull her pelvic floor in and up as much as possible (as if trying to stop urine from coming out) for 2–3 s. The researcher will observe the participant to ensure that she is breathing normally. Three squeezes will be recorded at intervals of 10 s between the efforts, and the average will be recorded in cms/H_2_O as the maximum perceived strength of the PFM; the higher the reading the higher the PFS [21].

The Incontinence QoL questionnaire is a valid and reliable questionnaire used to assess QoL with excellent internal consistency (Cronbach’s value of 0.93 and 0.79–0.89) and has no ceiling effect [43]. It is a 22 item self-reported disease-specific questionnaire that assesses the effect of UI on QoL under three (3) subscales of Avoidance and Limiting Behaviour (ALB), Psychosocial Impact (PSI), and Social Embarrassment (SE) [28, 43]. Specifically, the ALB subscale consists of 8 items, the PSI consists of 9 items, and the SE consists of 5 items. Moreover, scores ranging from 1 (extreme) to 5 (not at all) will be used to grade each of the items, and the total scores (for the 22 items) will then be transformed to a “scale score” ranging from 0 to 100 using the following formula:

$${\text{Scale}}\,{\text{score}} = {\text{Sum}}\,{\text{of}}\,{\text{the}}\,{\text{items}}{-}{\text{Lowest}}\,{\text{possible}}\,{\text{score/Possible}}\,{\text{raw}}\,{\text{score}}\,{\text{range }} \times 100.$$

Higher scores from all the items indicate less impact of PFRI on QoL [28, 43].

The Female Sexual Function Index (FSFI) is a questionnaire used in the assessment of female sexual function. The questionnaire possesses good psychometric properties (high test–retest reliability for each domain r = 0.79 to 0.86, and high internal consistency (Cronbach’s values of 0.82 and above) and it is easy to administer [44]. It is a multi-dimensional questionnaire with 19 items that are self-reported for assessing SF in women. It comprises six (6) domains: desire, subjective arousal, lubrication, orgasm, satisfaction, and pain. The desire domain has 2 items with scores ranging from 1 to 5 in each item, summed up to a total score of 2 (minimum) and 10 (maximum); the arousal domain has 4 items with scores ranging from 0 to 5 in each item, summed up to a total of minimum and maximum scores of 0 and 20 respectively; the lubrication domain has 4 items with scores ranging from 0 to 5 in each item, summed up to a total of minimum and maximum scores of 0 and 20 respectively; the orgasm domain has 3 items with scores ranging from 0 to 5 in each item, summed up to a total of minimum and maximum scores of 0 and 15 respectively; the satisfaction domain has 3 items with scores ranging from 1 to 5 in each item, summed up to a minimum and maximum score of 2 and 15 respectively; and the pain domain has 3 items with scores ranging from 0 to 5 in each item, summed up to a total of minimum and maximum score of 0 and 15 respectively. An FSFI total score of less than or equal to 26.55 indicates risk for sexual dysfunction.

The Hospital Anxiety and Depression rating scale (HADs) will be used to assess mental health (MH). It is a valid and reliable questionnaire used in determining the mental status of an individual [45]. Each participant will be asked to complete the HADs after clear and detailed explanation by the researcher on how to complete the questionnaire. The HADs comprises seven questions for anxiety and seven for depression and it takes 2–5 min to complete. The anxiety and depression questions are combined in the scale; however, it is recommended that they are scored separately [46]. Both scales demonstrate good to moderate to high internal consistency; Cronbach's alpha for anxiety varied from 0.68 to 0.93 (mean 0.83) and for depression from 0.67 to 0.90 (mean.82) [45].

## Data analysis

Microsoft Excel will be used to record all the data obtained before being exported to the Statistical Package for Social Sciences (SPSS). Descriptive and inferential statistics will be used to analyse the data. All study parameters will be summarized using descriptive statistics of frequency, mean, standard deviation, and percentage. Factorial ANOVA will be used to determine between-group differences/interactions in the study groups from the pre to the follow-up study period for PFS and UL among study participants. The Wilcoxon signed-rank test will be computed to determine differences between the pre, post and follow-up intervention for SF, ML and QoL scores in both the experimental and control groups. The independent t-test will be used to determine the difference in the post intervention PFS and UL scores between the experimental and control groups, while Mann–Whitney U will be used to determine the difference in the post intervention for SF, ML and QoL scores between the experimental and control groups. The magnitude of the differences between the groups will be determined by calculating the effect sizes. Missing data will be handled through multiple imputation. All analyses will be performed with probability set at 0.05 alpha level.

## Discussion

The Paula exercise method is a growing conservative method used in the management of urinary incontinence with significant effect in reducing urine leakage and improving pelvic floor muscles strength, QoL and SF as reported by some studies [23, 31, 33]. However, this intervention has not been tested on women with post-fistula-repair incontinence and the evidence regarding the use of alternative exercise other than PFMT for UI has been sketchy. Women with PFRI are devastated and many continence surgeries only worsen their conditions [28]. Therefore, there is an urgent need for investigation of other conservative approaches that may help the affected women to re-integrate back into the society.

The outcome of this study will provide evidence on whether PEM can be used in the management of PFRI. Also, it will further support the use of Paula exercise as a conservative treatment for UI. Findings shall be disseminated appropriately at relevant scientific forums.

## Data Availability

The data for this trial will be made available upon request.

## Abbreviations

VVF: Vesicovaginal fistula
PFRI: Post fistula repair incontinence
PFS: Pelvic floor strength
UI: Urinary incontinence
PFM: Pelvic floor strength
PFMT: Pelvic floor strength training
PEM: Paula exercise method
UL: Urine leakage
QoL: Quality of life
MH: Mental health
SF: Sexual function
FSFI: Female Sexual Function Index
HADs: Hospital Anxiety and Depression Rating Scale
ALB: Avoidance and limiting behavior
PSI: Psychosocial impact
SE: Social embarrassment
